# Sequevar Diversity and Virulence of *Ralstonia solanacearum* Phylotype I on Mayotte Island (Indian Ocean)

**DOI:** 10.3389/fpls.2017.02209

**Published:** 2018-01-05

**Authors:** Thomas Chesneau, Géraldine Maignien, Claudine Boyer, Jean-Jacques Chéron, Michel Roux-Cuvelier, Luc Vanhuffel, Stéphane Poussier, Philippe Prior

**Affiliations:** ^1^UMR PVBMT, CIRAD, Saint-Pierre, La Réunion, France; ^2^Etablissement Public National, Coconi, France; ^3^Union Interprofessionnelle Châtaigne Périgord - Limousin - Midi-Pyrénées, Tulle, France; ^4^Chambre d'Agriculture de la Pêche et de l'aquaculture de Mayotte, Saint Pierre, La Réunion, France; ^5^Chambre d'Agriculture de la Pêche et de l'aquaculture de Mayotte, Mamoudzou, France; ^6^UMR PVBMT, University of Réunion, Saint-Denis, France; ^7^UMR PVBMT, Institut National de la Recherche Agronomique, Saint-Pierre, France

**Keywords:** *Ralstonia solanacearum* phylotype I, sequevars, virulence, Mayotte, Indian Ocean

## Abstract

The genetic and phenotypic diversity of the *Ralstonia solanacearum* species complex, which causes bacterial wilt to Solanacae, was assessed in 140 strains sampled from the main vegetable production areas of the Mayotte island. Only phylotype I strains were identified in the five surveyed areas. The strains were distributed into the following 4 sequevars: I-31 (85.7%), I-18 (5.0%), I-15 (5.7%), and I-46 (3.6%). The central area of Mayotte was the most diverse region, harboring 4 sequevars representing 47.1% of the collected strains. Virulence tests were performed under field and controlled conditions on a set of 10 tomato breeding line accessions and two commercial hybrid tomato cultivars. The strains belonging to sequevar I-31 showed the highest virulence on the tomatoes (pathotypes T-2 and T-3), whereas sequevars I-18, I-15, and I-46 were grouped into the weakly T-1 pathotype. When the tomato accessions were challenged in the field and growth chambers, the highest level of resistance were observed from the genetically related accessions Hawaii 7996, R3034, TML46, and CLN1463. These accessions were considered moderately to highly resistant to representative strains of the most virulent and prevalent sequevar (I-31). Interestingly, the Platinum F1 cultivar, which was recently commercialized in Mayotte for bacterial wilt resistance, was highly or moderately resistant to all strains. This study represents the first step in the rationalization of resistance deployment strategies against bacterial wilt-causing strains in Mayotte.

## Introduction

The *Ralstonia solanacearum* species complex (RSSC) (Gillings and Fahy, 1994) is responsible for bacterial wilt on a broad range of plant hosts comprising more than 200 species in at least 50 families (Hayward, 1994). RSSC is particularly destructive for vegetable crops, including potato, tomato, eggplant and pepper plants. RSSC strains are known for their unusually broad genetic basis and phenotypic diversity in tropical and subtropical areas (Hayward, 1991). Soil-borne RSSC strains invade the roots and colonize the xylem vessels (Vasse et al., 1995), leading to wilt symptoms and the death of their hosts. RSSC strains have been frequently reported to develop latent infections that are maintained at high concentrations in asymptomatic hosts (Grimault and Prior, 1993). Breeding for resistance remains the most effective and sustainable strategy to control bacterial wilt (Prior et al., 1994). Unfortunately, resistance to bacterial wilt often breaks down due to the genomic plasticity and the large genetic and phenotypic diversity within RSSC (Gillings and Fahy, 1994; Lebeau et al., 2011). The sustainability of host resistance is tried to a large-scale local management strategy that includes study of the genetic diversity of bacterial wilt-causing strains and their virulence patterns (Lebeau et al., 2011).

Historically, RSSC strains have been classified into races and biovars based on their host ranges and biochemical properties (Buddenhagen et al., 1962; Hayward, 1964), but these classifications are neither predictive nor phylogenetically meaningful. Subsequently, strains unifying the RSSC were distributed into four major phylotypes of different geographical origins named phylotypes based upon phylogenetic analyses of sequence data generated from the 16S-23S internal transcribed spacer (ITS) region as follows: I from Asia, II from the America, III from Africa and the Indian Ocean, and IV from Australia, Japan, and Indonesia (Fegan and Prior, 2005). More recently, the RSSC was taxonomically organized into three species that classified phylotypes I and III as *R. pseudosolanacearum*, phylotype II as *R. solanacearum* and phylotype IV as *R. syzygii* (Safni et al., 2014; Prior et al., 2016). The phylotypes are subdivided into sequevars based on sequence variation in the endoglucanase (*egl*) partial gene (Fegan and Prior, 2005).

Mayotte is a small island located in the southwest Indian Ocean, more precisely in the Comoros archipelago between Eastern Africa and Madagascar. Small-scale farming systems have been developed, especially with vegetable production, which strongly contributes to secure agricultural resources. From the panel of tropical plant diseases, the severity of bacterial wilt outbreaks is the major constraint to vegetable production, especially during the warm season. Tomatoes are largely consumed throughout the year; therefore, improvement of tomato production during the off-season (corresponding to the rainy and wet season) remains highly strategic for farmers income. In Mayotte, bacterial wilt has been recognized for a long time on tomato (*Solanum lycopersicum*), eggplant (*Solanum melongena*), sweet pepper (*Capsicum annuum*), hot pepper (*C. frutescens*) and European black nightshade (*Solanum nigrum*) plants. Various unpublished studies have also been undertaken during the last two decades to assess the resistance of tomato and eggplant varieties against local RSSC strains. As a secluded island in the Comoros archipelago, Mayotte is located in a strategically poor documented area that covers the spectrum of RSSC diversity.

The literature available on the distribution and economic importance of RSSC in Africa and the Indian Ocean remains discrete (Elphinstone, 2005), although broad diversity has been reported, with three of the four known phylotypes identified. In African countries where large samplings have been achieved, such as Cameroon, the Ivory Coast and Ethiopia, phylotypes I and IIA have been reported to be more prevalent in the lowlands, whereas phylotypes IIB-1 and III are mostly observed in the highlands (Lemessa and Zeller, 2007; Mahbou Somo Toukam et al., 2009; N'Guessan et al., 2012). Broad genetic diversity of phylotype III strains has been reported in a few countries in Sub-Saharan Africa (Angola, Burkina Faso, Cameroon, Guinea, the Ivory Coast, Kenya, and Zimbabwe) and the southwest Indian Ocean (Madagascar and Reunion) (Ravelomanantsoa et al., 2016).

In this study, we investigated an almost closed and uniform agronomical landscape in which we assumed that the RSSC population was well-established due to its insularity and limited international exchanges compared to continental environments. A 140-strain collection was sampled, which allowed us to unravel the genetic diversity of RSSC and assign the phylogenetic positions of the strains. As a prerequisite to understanding, extending and managing the success of commercially resistant tomato cultivars, the virulence of a subset of representative strains was assayed using resistant tomato breeding lines under field and controlled conditions.

## Materials and methods

### Bacterial strains

**Figure 1:**
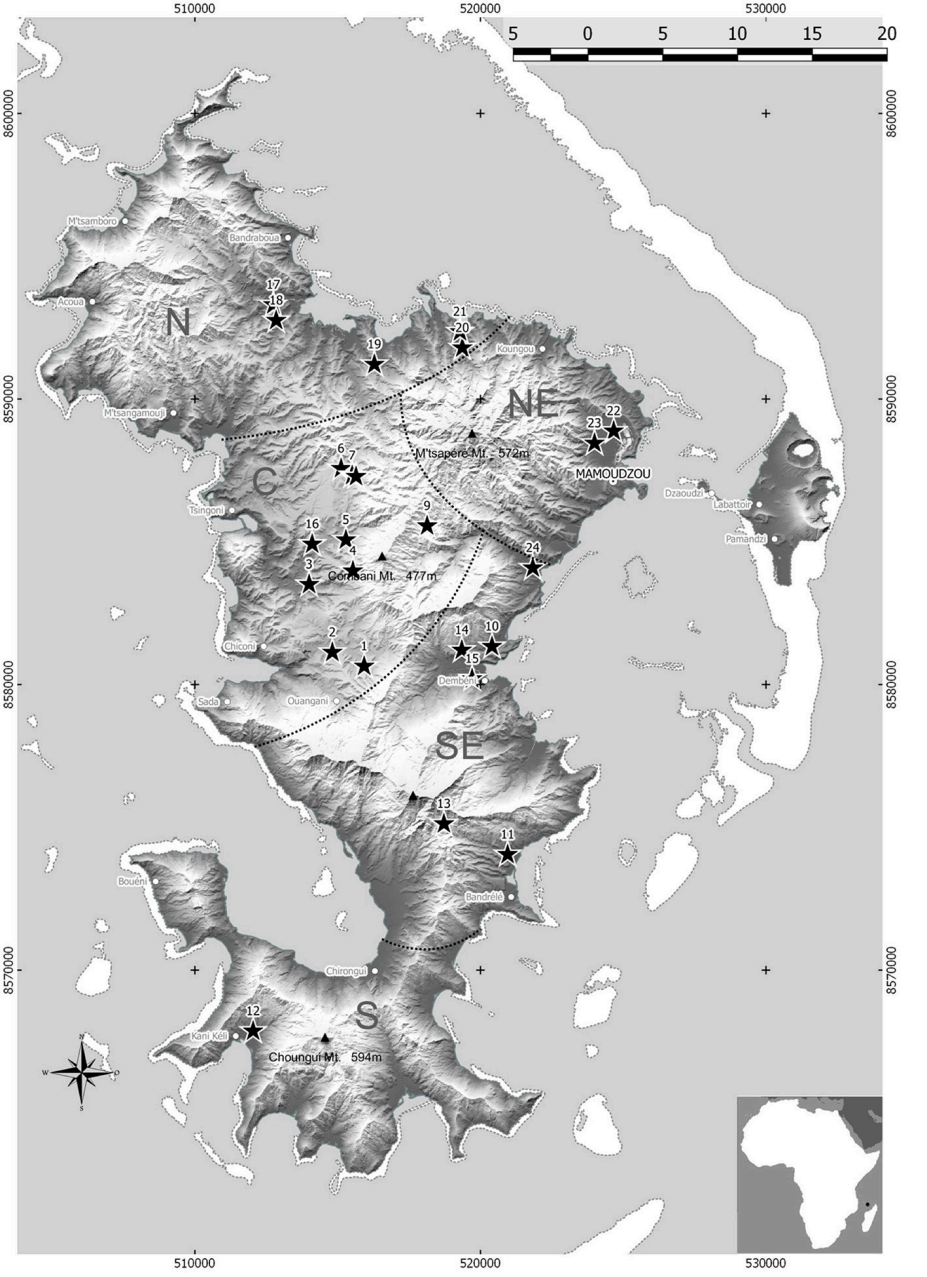
Mayotte map showing the five main vegetable areas (C, Centre; N, North; NE, Northeast; S, South; SE, Southeast) and the 24 sites where phylotype I strain sequevars 31, 18, 46, and 15 were collected.

Although Mayotte is a small island (20 × 40 km), the main vegetable production areas are distributed into five agro-ecological zones (Figure 1). Bacterial wilt occurred in all areas surveyed in September 2012. Solanaceous plants (tomato, eggplant, hot pepper, sweet pepper, and black nightshade) showing typical bacterial wilt symptoms were sampled at 24 sites with a particular emphasis on the tomato, which is highly susceptible to bacterial wilt in Mayotte. For each plant, a large stem segment (5–10 cm in length) was cut and maintained at approximately 25°C prior to bacterial isolation and purification. The stems were surface-disinfected with 70% ethanol, and a sub-fragment (0.5 cm) was shredded and macerated in sterile distilled water to allow bacterial release. Then, the macerates (50 μL) were individually streaked onto tetrazolium chloride (TZC) agar medium (Kelman, 1954) for 48–72 hours at 28°C. For each sample, one typical RSSC colony was re-streaked onto new TZC medium for further purification and bacterial species validation. Finally, each strain was assigned a CIRAD Reunion (RUN) identification number (Table S1) and maintained at −80°C on Cryobank® microbeads at Cirad (Saint-Pierre, Reunion).

### Phylogeny based on the endoglucanase (*egl*) partial gene

The endoglucanase partial gene was sequenced from 140 strains to identify their phylotypes and sequevars and to create a phylogenetic tree. A 750-bp fragment of the *egl* gene was amplified using the Endo-F /Endo-R primer pair as previously reported (Cellier and Prior, 2010). The PCR products were dehydrated in a vacuum and sent to Beckman Coulter Genomics (Takeley, UK) for further purification and double-strand sequencing using the PCR primers as the sequencing primers. Raw sequences from both strands were edited, trimmed and aligned under the ARB software package (http://www.arb-home.de/) (Ludwig et al., 2004). The sequences were trimmed starting at 5′-ACGGCGAT-3′ and ending at 5′-ACGGCGGC-3′. A phylogenetic tree was constructed with the 140 isolated strains from Mayotte together with a set of international reference strains (Table S2) using the neighbor-joining method (Saitou and Nei, 1987) with 5,000 bootstrap resampling runs to test the robustness of the tree topology. Assignment to *egl*-based sequevars was conducted using reference *egl* sequences (Prior and Fegan, unpublished data). The sequences of the newly described strains were deposited into the GenBank database under accession numbers MF359095 - MF359234 (Table S1).

### Evaluation of tomato resistance to mayotte phylotype I

First, we tested the resistance properties of 10 tomato accessions (Core collection) that were internationally considered references for tomato resistance origins (Table S3) (Lebeau et al., 2011). The field assay was conducted in a field located at Dembeni (Southeastern Mayotte), which is an area that is naturally infested with sequevar I-31 (the most prevalent sequevar in Mayotte; this study). This experiment was conducted under a full ground tunnel during the hot and wet season that was favorable for the development of bacterial wilt. This assay was performed using a random experimental plan of four repetitions corresponding to four blocks of 10 plants per modality defined as following:CRA66 (T1), Okitsu Sozai no.1 (T2), NC 72 TR 4-4 (T3), IRAT L3 (T4), Hawaii 7996 (T5), TML46 (T6), CLN1463 (T7), R3034 (T8), L285 (T9), and L390 (10). The plantlets were grown under nursery conditions and planted at the three to four fully expanded leaf stage in double lines with a density of 3 plants per m2. The water supply was provided by a drip system using a pump equipped with two sand filters, a disk filter and a pressure regulator. Disease progression was visually assessed weekly over a 2-month period by marking each plant as “dead” or “not dead” (all leaves wilted).

A second virulence test was conducted at Cirad Reunion in growth chambers (Rotoplan) under a routine security norm level (NS2). Six tomato lines of the Core collection (Lebeau et al., 2011) were chosen based on the field assay results. Two commercial tomato hybrid F1 cultivars (Cobra and Platinum) were added to this test, since they were widely cropped in Mayotte (Table S3). All of these accessions were challenged with a subset of 8 strains representative of the genetic diversity of RSSC from Mayotte (Table 1). The strains were chosen according to their phylotype-sequevar classification, geographical location and isolation host. Two strains were selected from each of the 4 sequevars identified. The sequevar I-31 strains RUN2108 and RUN2170 were selected since they were isolated at the “Station Agronomique de Dembeni” and the “Lycée Agricole de Coconi,” which are the main experimental sites for vegetable production in Mayotte. Additionally, the RUN2108 strain was isolated in the experimental full ground tunnel used for the tomato field assay. Virulence was tested on plantlets with 3–4 fully expanded leaves. Three repetitions of 10 pots with one healthy plantlet with three to four true leaves were selected for each plant x strain combination. The strains were grown at 30°C on Kelman solid medium with 0.5 g of yeast extract (Kelman, 1954). Bacterial suspensions calibrated to 5 × 10^8^ colony-forming units were prepared as previously described (Cellier and Prior, 2010). Inoculation was achieved by pouring 2 mL of inoculum on lateral roots previously wounded with a scalpel. All plants were placed in growth chambers with a 12-h photoperiod, 25 ± 2°C (night) and 30 ± 2°C (day) temperature, and 80% relative humidity. Bacterial wilt development was monitored for 15 days after inoculation by scoring the number of wilted plants as follows: asymptomatic (no symptoms), wilting plant (at least one wilted leaf) or dead (all wilted leaves) (Lebeau et al., 2011). After the final record, the remaining healthy plants were tested to determine whether they were carrying a latent infection. For each plant, a cutting 2 to 3 cm in length at the stem base was immersed in 5 mL of Tris buffer solution at room temperature for 2–3 h to allow bacterial release. A 50-μL aliquot of each extract was streaked onto semi-selective modified Granada and Sequeira medium (Poussier et al., 2002) and incubated for 72 h at 28°C. Latent infections were scored as positive or negative depending on the development of colonies on the plates. A colonization index was calculated for each plant x strain combination according to the formula CI = N_wp_ + (N_s_ × R_s_), where N_wp_ was the percentage of wilted plants, N_s_ was the percentage of asymptomatic plants and Rs was the percentage of asymptomatic plants with a latent infection (Lebeau et al., 2011).

**Table 1:** *Ralstonia solanacearum* phylotype I strains from Mayotte selected for the virulence test against tomato accessions under controlled conditions.

**Strains**	**Location (area)**	**Isolation host**	**Species**	**Sequevar**
RUN2170	Coconi (C)	Eggplant	*Solanum melongena*	31
RUN2108	Dembeni (SE)	Tomato	*Solanum lycopersicum*	31
RUN2083	M'romouhou (SE)	Hot pepper	*Capsicum annuum*	18
RUN2150	Miangani (N)	Tomato	*S. lycopersicum*	18
RUN2127	Combani (C)	Sweet pepper	*C. annuum*	46
RUN2146	Miangani (N)	Tomato	*S. lycopersicum*	46
RUN2143	Mitséni (N)	Sweet pepper	*C. annuum*	15
RUN2140	Mitséni (N)	Eggplant	*S. melongena*	15

### Data analysis

Effect of accessions on resistance phenotypes was tested by analysis of variance on the wilted rate means 120 days after plantation. The data was transformed with arcsine square root function to fit normal distribution of the variable. Then means differentiation between accessions was done using a pairwise comparison test by Tukey's “Honest Significant Differences” method (package agricolae, R statistical freeware software, version 3.4.2).

The strain phenotypes were scored according to the pathotypes as defined previously by testing the CoreRs2 (RSSC strain collection) on the Core-TEP collection (Lebeau et al., 2011). A pathotype was defined as a group of strains with similar virulence profiles on reference accessions of a host species, such as the tomato (Lebeau et al., 2011). A clustering approach was used to determine the phenotype scores according to the final wilting incidence and the colonization index of each accession-strain combination used in the study. The phenotype scores of each accession-strain combination were assigned using the “k-nearest neighbors” algorithm of the package *class* of the R statistical freeware (version 3.0.2) (Venables and Ripley, 2002) to the reference phenotype score classification, which comprised five levels: 1 = highly resistant, 2 = moderately resistant, 3.1 = partially resistant, 3.2 = latently infected, 4 = moderately susceptible, and 5 = highly susceptible (Lebeau et al., 2011). An agglomerative hierarchical nesting classification using the package *agnes* confirmed by a fuzzy clustering analysis using the package *cluster* was undertaken independently of the Core-Rs2 to identify tomato pathotypes for the 8 tomato accessions used for the study (Lebeau et al., 2011).

## Results

### Bacteria isolation and identification

A total of 140 RSSC strains were sampled from the 24 surveyed sites throughout the 5 vegetable cropping areas in Mayotte. Strains were collected from five Solanaceous crops at rates of 57.1% for tomato (*n* = 80), 32.9% for eggplant (*n* = 46), 5.0% for sweet pepper (*n* = 7), 2.1% for hot pepper (*n* = 3), and 2.9% for black nightshade (*n* = 4).

### Phylotype and sequevar phylogeny

**Figure 2:**
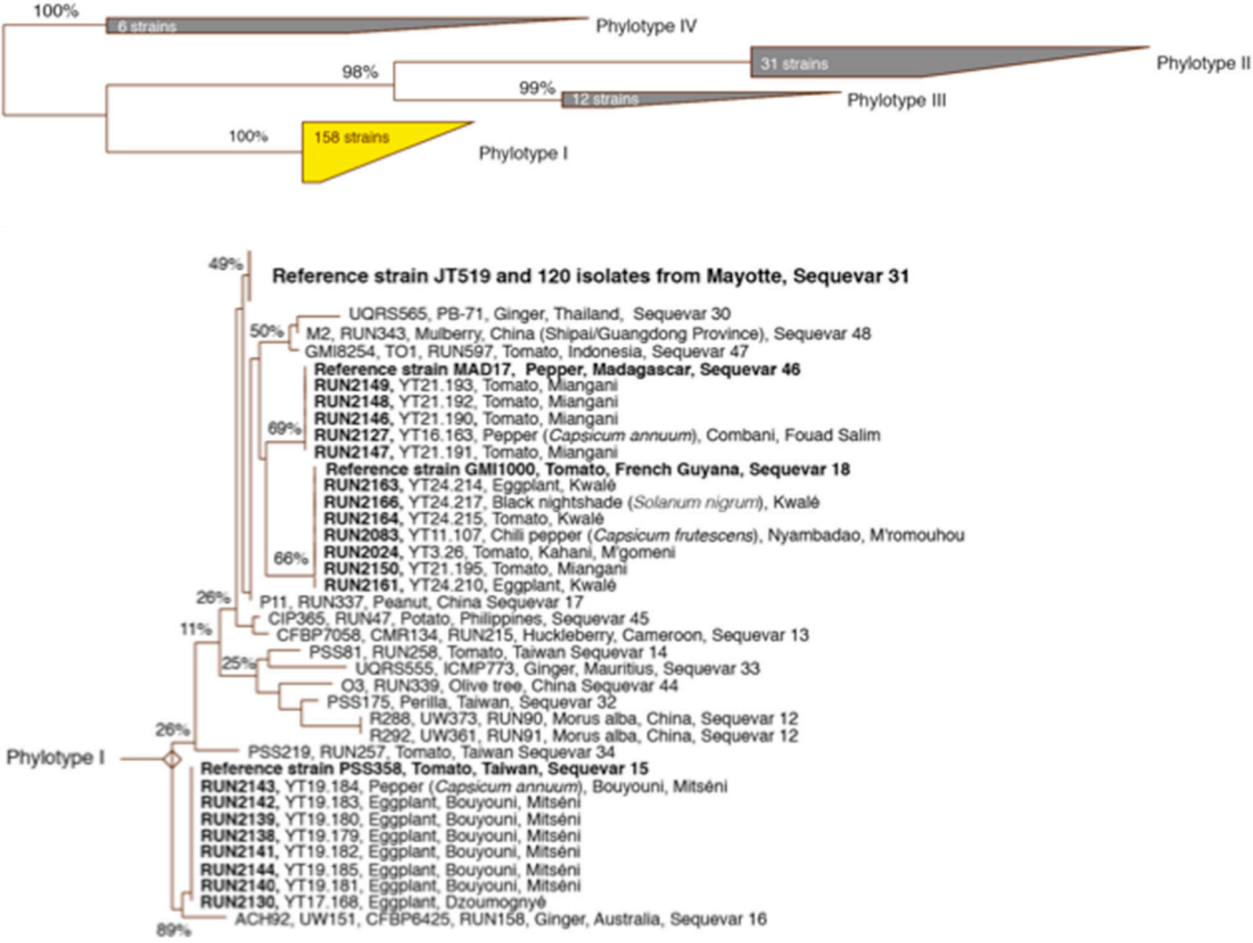
Phylogenetic neighbor-joining tree based on partial endoglucanase (*egl*) gene sequences from strains from Mayotte and reference RSSC strains. The number localized at each node is the bootstrap value (*n* = 5,000), with significance <100% indicated at each node. The scale bar represents 1/100 nucleotide substitutions.

Only phylotype I strains were identified according to the most recent RSSC taxonomy (Safni et al., 2014; Prior et al., 2016). The sequevar assignation of the strains based on *egl* sequencing (140 strains) revealed the presence of four sequevars (I-15, I-18, I-31 and I-46) (Figure 2). The prevalence and distribution of the four sequevars appeared to be variable throughout the five vegetable production areas in Mayotte (Table S4). Sequevar I-31 (*n* = 120) was the most prevalent, representing 85.7% of the collection compared to sequevars I-18 (*n* = 7), I-15 (*n* = 8), and I-46 (*n* = 5), which represented 5.0, 5.7, and 3.6% of the collection, respectively. Sequevar I-31 was isolated from the northern area where 15.7% of the strains were collected (*n* = 22). Sequevars I-18 and I-15 were isolated from the central and southeastern areas, whereas I-46 was detected only in the central area. Only one sequevar (I-31, *n* = 17) was identified in the northeastern area. The central area is the most important vegetable production area in Mayotte. Sequevar diversity was the highest in this area. Of the 4 identified sequevars, sequevar I-31 (*n* = 48) represented 72.7% of the diversity, followed by the remaining strains, which belonged to sequevars I-18 (9.1%, *n* = 6), I-46 (7.6%, *n* = 5), and I-15 (10.6%, *n* = 7). Sequevar I-31 also predominated in the south and southeastern areas where it represented 100.0% (*n* = 14) and 90.5% (*n* = 19) of the collected strains, respectively. In the latter area, sequevars I-18 and I-15 were also identified and represented 4.8% (*n* = 1) of the diversity each. The prevalence of the four sequevars appeared to be variable depending on the host species in Mayotte (Table S5). Sequevar I-31 was consistently isolated and was predominant on all sampled host species, followed by I-18 on four host species and I-46 and I-15 on 2 host species each. Sequevars I-31, I-18, and I-46 were isolated on tomatoes with frequencies of 91.3% (*n* = 73), 3.8% (*n* = 3), and 5.0% (*n* = 4), respectively. Additionally, three sequevars each were identified on sweet pepper [I-31 (71.4%, *n* = 5), I-46 (14.3%, *n* = 1), and I-15 (14.3%, *n* = 1)] and eggplant [I-31 (80.4%, *n* = 37), I-18 (4.3%, *n* = 2), and I-15 (15.2%, *n* = 7)]. Two sequevars each were identified on hot pepper and black nightshade, with I-31 and I-18 representing 66.7% (*n* = 2) and 75.0% (*n* = 3) of the identifications on the hot peppers and 33.3% (*n* = 1) and 25.0% (*n* = 1) of the identifications on the black nightshade, respectively. A neighbor-joining tree based on partial *egl* sequences allowed us to determine the phylogenetic positions of the 140 Mayotte strains compared with the worldwide reference strains of RSSC phylotypes I (18 strains), II (31 strains), III (12 strains), and IV (6 strains) (Figure 2). The 120 sequevar I-31 strains isolated in Mayotte were phylogenetically identical to the reference sequevar I-31 strain JT519 from La Réunion. The eight sequevar I-15 strains and the five sequevar I-46 strains isolated in Mayotte appeared to be phylogenetically identical to the reference sequevar I-15 and I-46 strains PSS358 and MAD17 from Taiwan and Madagascar, respectively. The seven sequevar I-18 strains isolated in Mayotte were phylogenetically closely related to the reference sequevar I-18 strain GMI1000 from French Guiana.

### Tomato resistance to phylotype I in mayotte

**Figure 3:**
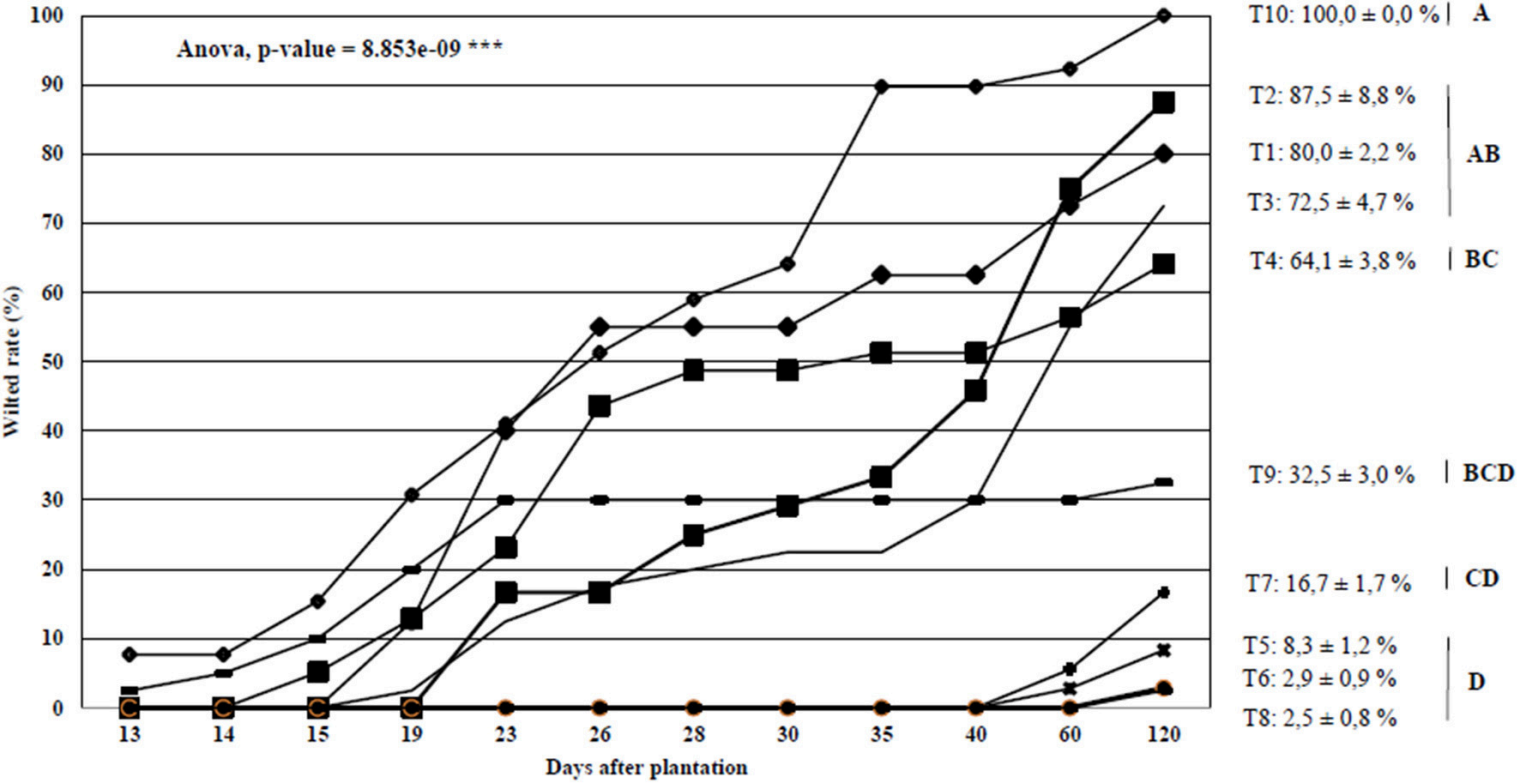
Bacterial wilt progression curves on 10 Core tomato accessions screened under field conditions at the Dembeni experimental station during the 2011 hot and wet season. Means and standard errors are shown. Homogeneous groups were created transforming wilted datas with arcsin square root function and performing variance analysis followed by a Tukey's HSD test.

Bacterial wilt progression on 10 tomato accessions was followed for 2 months in a field naturally contaminated with sequevar I-31, which was the most prevalent sequevar in Mayotte. The statistical analysis (allowed us to separate the 10 tomato accessions into six groups (Figure 3). The first goup included the most susceptible control T10 with 100.0% of wilted rate. The second group comprised susceptible accessions tomatoT2, T1, and T3, which showed 87.5, 80, and 72.5%, wilted plants, respectively, at the end of the experiment. The third group was composed of one tomato accession (T4) that showed 64.1% of wilted plants. The fourth group included also one accession, T9 that appeared to be moderately resistant, with 32.5% wilted plants. The fifth group was resistant with one accession T7 showing 15.0% wilted plants. The last group was composed of the three most resistant tomato accessions (T5, T6, and T8), resulting in 8.3, 2.9, and 2.5% wilted plants, respectively. Considering these results, the resistance properties of the six tomato accessions (T4, T5, T7, T8, T9, and T10) belonging to five groups were further evaluated under controlled conditions.

The virulence of 8 strains (2 strains each of sequevar I-15, I-18, I-31, and I-46) representing phylotype I sequevar diversity in Mayotte (YT) were tested on 6 tomato accessions and 2 tomato cultivars that were widely cropped in Mayotte (Table 2). Interestingly, the mean wilting rate of the six tomato accessions used in the field and the growth chamber assays appeared to be very similar (Table S6). The susceptible control accession T10 scored as highly susceptible to all tested strains [wilting incidence (= W) between 100 and 76.7% and colonization index (= CI) between 100 and 75%], even though this accession was slightly more affected by the two sequevar I-18 strains (RUN2083 and RUN2150; W = 30.0 and 53.3% and CI = 35.3 and 53.3%, respectively). Higher levels of resistance were observed with T5 (W from 0 to 10% and CI from 0 to 25.3%) and T8 (W from 0 to 8.1% and CI from 0% to 26.7%). T5 was classified as highly resistant (phenotype score *P* = 1) to 6 strains and moderately resistant (*P* = 2) to 2 strains. T8 was classified as highly resistant to 5 strains and moderately resistant to 3 strains. The commercial hybrid cultivar Platinum appeared to be more resistant than the commercial hybrid cultivar Cobra. The Platinum cultivar was classified as highly resistant to 4 strains (I-15, I-18, and I-46) and moderately resistant to 4 strains (I-15, I-31, and I-46), whereas the Cobra cultivar was classified as highly resistant to two strains (I-18), moderately resistant to four strains (I-15 and I-46), partially resistant to one strain (I-31) and moderately susceptible to one strain (I-31).

**Table 2:** Phenotypic responses of six tomato accessions and two commercialized tomato cultivars inoculated with the Core-RsYT under controlled conditions in La Reunion and compared with reference data from the Core-Tep/Core-Rs2.

**Accessions**^**a**^	***R. pseudosolanacearum* strain*s* from Mayotte (phylotype-sequevar)**^**b**^																															
	**RUN2170 I-31**				**RUN2108 I-31**				**RUN2083 I-18**				**RUN2150 I-18**				**RUN2127 I-46**				**RUN2146 I-46**				**RUN2143 I-15**				**RUN2140 I-15**			
	**W**	**CI**	**P**	**C**	**W**	**CI**	**P**	**C**	**W**	**CI**	**P**	**C**	**W**	**CI**	**P**	**C**	**W**	**CI**	**P**	**C**	**W**	**CI**	**P**	**C**	**W**	**CI**	**P**	**C**	**W**	**CI**	**P**	**C**
C	30.0	46.4	3.1	3	56.7	68.2	4	6	0.0	3.0	1	1	0.0	13.7	1	1	10.0	23.2	2	2	10.0	24.8	2	2	30.0	41.6	2	3	10.0	17.0	2	2
P	20.0	36.0	2	3	23.3	39.0	2	3	0.0	13.7	1	1	0.0	5.3	1	1	**0.0**	13.7	1	1	13.3	26.2	2	2	6.7	24.4	2	2	3.3	11.7	1	1
T4	58.5	51.2	4	5	56.0	70.3	4	6	5.6	18.3	2	1	6.7	12.9	1	1	3.3	17.3	1	1	10.0	26.4	2	2	30.0	41.6	2	3	21.1	29.3	2	3
T5	**0.0**	15.3	1	1	10.0	25.3	2	2	**0.0**	**0.0**	1	1	**0.0**	**0.0**	1	1	**0.0**	8.0	1	1	**0.0**	8.3	1	1	6.7	14.5	1	1	10.0	16.6	2	2
T7	16.7	38.5	2	3	16.7	27.3	2	2	**0.0**	**0.0**	1	1	10.0	18.6	2	2	3.3	19.8	2	1	13.3	20.7	2	2	**0.0**	11.0	1	1	6.7	16.3	2	1
T8	8.1	26.7	2	2	3.3	22.4	2	1	**0.0**	**0.0**	1	1	**0.0**	8.3	1	1	**0.0**	9.0	1	1	**0.0**	11.0	1	1	**0.0**	20.3	2	1	**0.0**	8.0	1	1
T9	26.7	39.5	2	3	40.0	53.3	3.1	4	6.7	13.0	1	1	3.3	6.3	1	1	16.7	40.6	2	3	**0.0**	14.0	1	1	20.0	30.0	2	3	26.7	37.1	2	3
T10	93.3	90.0	5	8	100.0	100.0	5	8	30.0	35.3	2	3	53.3	53.3	4	5	100.0	100.0	5	8	76.7	88.6	4	7	96.7	90.0	5	8	83.3	75.0	4	7
Pathotype	type T-2				type T-3				type T-1				type T-1				type T-1				type T-1				type T-1				type T-1			

a *Tomato accessions. T, pathotype on tomato; C, Cobra F1; P, Platinum F1; Path, Classification of Mayotte tomato pathotypes compared to those of the tomato Core-tep/CoreRs2*.

b *W, wilted plants (%); CI, colonization index (%); P, phenotype; C, Mayotte classification, identified using the k-nearest neighbors classification with reference to the Core-Tep for P and Prof, the tomato pathotype from the Core-tep/CoreRs2 for Path and by agglomerative hierarchical nesting classification and the average linkage method for C. P scale: 1, highly resistant; 2, moderately resistant; 3.1, partially resistant, 3.2, latent infection; 4, moderately susceptible; and 5, highly susceptible (Lebeau et al., 2011)*.

The eight strains were separated into three clusters by referring to previously defined pathotype typing (Lebeau et al., 2011) (Table 2). The first cluster encoded T-1 and grouped the less virulent strains belonging to sequevars I-15, I-18, and I-46. Clusters T-2 and T-3 comprised the more virulent strains RUN2170 and RUN2108 that belonged to the sequevar I-31 strains. Strain RUN2108 isolated at the Dembeni site was highly virulent to the 6 tomato lines and the commercial cultivars used during this study. An agglomerative hierarchical nesting classification was used to distinguish the phenotypic variability of the strains on the tomato lines and cultivars used in this study. Two clusters ranked as types YT-1 and YT-2 were identified. Again, the two sequevar I-31 strains scored with the highest virulence and were typed as YT-2, whereas strains I-18 and I-46 were assigned to the weakest virulence type YT-1. The sequevar I-15 strains were distributed in YT-1 (RUN2140) and YT-2 (RUN2143).

## Discussion

The RSSC strains are distributed into four monophyletic clusters of strains termed phylotypes based on a hierarchical classification scheme. These phylotypes are further subdivided into sequevars based on polymorphisms of the endoglucanase gene (*egl*) (Fegan and Prior, 2005). RSSC was reported to be broadly diverse in most subtropical and tropical areas where wide sampling and genetic diversity studies were performed. For example, in Africa, three phylotypes (I, II, and III) were identified in Cameroon and the Ivory Coast (Mahbou Somo Toukam et al., 2009; N'Guessan et al., 2012), and two phylotypes (I and II) were identified in Ethiopia (Lemessa and Zeller, 2007). In Asia, three phylotypes (I, II, and IV) were reported in India (Sagar et al., 2014), and two phylotypes (I and IV) were identified in Japan (Horita et al., 2014) and (I and II) China (Xu et al., 2009).

In this study, we assessed the phylogenetic diversity of RSSC in Mayotte. We found that the phylogenetic diversity was highly homogeneous, since only phylotype I was identified (*R. pseudosolanacearum*) according to the most recent RSSC taxonomy (Safni et al., 2014; Prior et al., 2016). Phylotype I is the most prevalent phylotype in other southwest Indian Ocean islands (SWIO), such as Comoros, Mauritius, Reunion, Rodrigues, and Seychelles, where it accounts for 87% of the strain phylogenetic diversity and is mainly isolated from solanaceous crops (Yahiaoui et al., 2016a). Phylotype I has also been reported in Madagascar and eastern African countries bordering the Indian Ocean, such as Kenya and South Africa (Wicker et al., 2012; Carstensen et al., 2016; Ravelomanantsoa, 2016). Phylotype I affects a wide range of crops that include both herbaceous and woody plants (Hayward, 1994), is distributed worldwide (Hayward, 1991) and is reported to be highly recombinogenic (Coupat et al., 2008; Wicker et al., 2012). Phylotype I is known for its broad infrasubspecific diversity and comprises 16 out of the 57 sequevars that are currently known. In Mayotte, we identified four sequevars (I-15, I-18, I-31, and I-46) of which sequevar I-31 had a high prevalence (85.7%). Interestingly, a similar situation was reported in another island in Taiwan, where only phylotype I was identified based on a 58-strain collection with the exception of two introduced *R. solanacearum* phylotype IIB-1 isolates (Lin et al., 2014). However, in Taiwan, the intraspecific diversity was different and was much higher than the diversity in Mayotte, since 10 sequevars were identified (Lin et al., 2014) and sequevar I-15 was the most prevalent (60.7%). Importantly, although the strains in Mayotte were isolated only from solanaceous plants, the strains in Taiwan were isolated from 22 host species ranging from annual herbaceous plants to perennial woody plants, with solanaceous and non-solanaceous species representing 64.2 and 35.8% of the collected strains, respectively. These results obtained in Mayotte and Taiwan on solanaceous and non-solanaceous crops emphasize the importance of host species as a motor driving RSSC genetic diversity. This finding strongly suggests that futures studies focusing on RSSC genetic diversity should take into account host diversity by collecting RSSC from both agro- and natural ecosystems.

In Mayotte, we found that the most prevalent sequevar was sequevar I-31, which represented 85.7% of sequevars collected. Our study showed that the I-31 strains from Mayotte were phylogenetically identical to the sequevar I-31 strains from La Reunion. I-31 has also been reported in other southwest Indian Ocean islands, such as Comoros, Mauritius, Reunion, Rodrigues, and Seychelles (Yahiaoui et al., 2016a,b), as well as in Brazil (Rodrigues et al., 2012), and some African countries, such as the Ivory Coast (N'Guessan et al., 2012), Democratic Republic of Congo, Uganda, South Africa (Carstensen et al., 2016), Benin (Sikirou et al., 2015), and Kenya (unpublished data). Interestingly, similar to the situation in Mayotte, sequevar I-31 was also the most widespread and prevalent sequevar in countries where extensive RSSC surveys were conducted, including the Ivory Coast (N'Guessan et al., 2012) and southwest Indian Ocean islands (Yahiaoui et al., 2016a). The higher prevalence of I-31 strains may be explained by their higher virulence. I-31 strains have the capacity to infect a wide host range. In Mayotte, I-31 strains were isolated from the 5 solanaceous plants sampled (tomato, eggplant, hot pepper, sweet pepper, and black nightshade). In other countries, these strains have been isolated on both herbaceous plants (*Solanaceae, Geraniaceae, Begoniaceae*, and *Asteraceae*) (N'Guessan et al., 2012; Rodrigues et al., 2012; Yahiaoui et al., 2016a,b); unpublished data) and woody plants (Eucalyptus) (Carstensen et al., 2016). Moreover, in the Ivory Coast, I-31 strains were characterized by a broad virulence spectrum and high virulence level on all tomato and eggplant accessions tested (except two eggplant accessions) and were assigned to the two highest virulence pathoprofiles (e and f) defined by Lebeau et al. (2011). In Mayotte, the sequevar I-31 strains were clustered into the most virulent pathotypes T-2 and T-3 referring to the classification of Lebeau et al. (2011) when tested on tomatoes, whereas strains of sequevars I-18, I-46, and I-15 were clustered into the least virulent pathotype T-1. Under field and growth chamber conditions, Hawaii 7996 (T5) and R3034 (T8) showed the highest levels of resistance amongst all tomato breeding lines tested against the sequevar I-31 strains and the strains belonging to the three other sequevars (I-15, I-18, and I-46) uncovered in Mayotte. In the Ivory Coast, Hawaii 7996 (T5) and R3034 (T8) were also the best genitors against phylotype I and IIA strains even though their resistance was overcome by strains from two agroecological zones (N'Guessan et al., 2012). These results are consistent with the virulence tests conducted with the Core-Rs2 collection, which comprised global strains from phylotypes I, IIA, IIB, and III (Lebeau et al., 2011).

Interestingly, in Mayotte, the most resistant genitors [Hawaii 7996 (T5), R3034 (T8), CLN1463 (T7), and TML46 (T6)] to the four sequevars (I-15, I-18, I-31, I-46) were selected by AVRDC in Taiwan. Sequevars I-15 and I-18 but not sequevars I-31 and I-46 were also reported in Taiwan (Lin et al., 2014), indicating that these genitors were effective against strains not found in Taiwan. In Mayotte, the commercial hybrid Platinum F1 was classified as highly or moderately resistant depending on the strain tested, suggesting that resistance in this hybrid likely came from the AVRDC breeding lines. The other commercial hybrid (Cobra F1) was not resistant, particularly against the I-31 strains, strongly suggesting that its deployment in Mayotte was considered riskier and required the implementation of additional control measures against bacterial wilt. Altogether, these results represent a real incentive for plant breeders to use resistant tomato breeding lines and the commercial hybrid Platinum as genetic resources to create and deploy adapted cultivars in Mayotte.

This study is the first report linking RSSC virulence to phylotype I genetic diversity in Mayotte. This study represents an important starting point for regional breeding programmes for bacterial wilt resistance and deployment strategies not only in Mayotte but also in neighboring southwest Indian Ocean islands and African countries, where the I-31 sequevar is widespread. To understand the epidemiological and evolutionary relationships associated with this genetic lineage, population structure studies must be conducted using methods such as multilocus sequence typing (MLST) and multilocus variable number tandem repeat analysis (MLVA), which have been developed to study RSSC populations on different scales (from cropping areas to continents) (Wicker et al., 2012; N'Guessan et al., 2013; Parkinson et al., 2013; Ravelomanantsoa et al., 2016; Guinard et al., 2017).

## Conclusion

This is the first study of RSSC epidemiology performed in a small isolated island. Low phylogenetic diversity was identified with only Phylotype I strains collected on solanaceous crop. The absence of cross interactions with phylotype II and III strains provide a unique environment to better understand phylotype I plant resistance properties from worlwide breeding projects.

## Author contributions

PP, SP, MR-C, and TC: designed the study; LV, TC, and PP: prospected the strains; CB, J-JC, and TC: performed the microbiology and molecular biology experiments; GM and TC: conducted the phenotyping experiments; TC: drafted the manuscript; PP, MR-C, and SP: contributed to writing, drafting and revising the manuscript. All authors approved the final manuscript.

### Conflict of interest statement

The authors declare that the research was conducted in the absence of any commercial or financial relationships that could be construed as a potential conflict of interest.

## Supplementary material

Table S1 *Ralstonia solanacearum* strains isolated from Mayotte Island. ^a^Mayotte location and area of strain isolations (C, Centre; N, North; NE, Northeast; S, South; SE, Southeast). ^*^Strain used for the virulence tests.

Table S2 RSSC reference strains used in this study to structure the phylogenetic tree.

Table S3 Tomato accessions used for the virulence tests.

Table S4 Sequevar distributions of the *Ralstonia solanacearum* phylotype I strains within the five vegetable production areas of Mayotte.

Table S5 Sequevar distributions of the *Ralstonia solanacearum* phylotype I strains within the different host species sampled in Mayotte.

Table S6 Comparison table of the mean wilted rate of several tomato lines from the Core collection measured under field and controlled conditions against *Ralstonia solanacearum* phylotype I-31 strains from Mayotte. ^a^Tomato accessions from the tomato Core-Tep. ^b^Field experiment was assessed on the tomato Core-tep in Mayotte in 2011 and screened against strains characterized as *R. pseudosolanacearum* phylotype I sequevar 31. Notably, the RUN2108 (*Solanum lycopersicum*), RUN2116 (*Capsicum annuum*), and RUN2121 (*Solanum nigrum*) strains were collected in the same tunnel in 2012. ^c^Screening results obtained for strain RUN2108 (I-31), which was collected in the same tunnel in 2012 against several tomato Core-Tep lines under controlled conditions in La Reunion in 2013.
