# Supplementary material for: Sequevar Diversity and Virulence of Ralstonia solanacearum Phylotype I on Mayotte Island (Indian Ocean)
Source: Front Plant Sci. 2018 Jan 5;8:2209. doi: 10.3389/fpls.2017.02209 (PMC5760537; doi:10.3389/fpls.2017.02209)
Supplement: Table S1 — Ralstonia solanacearum strains isolated from Mayotte Island. aMayotte location and area of strain isolations (C, Centre; N, North; NE, Northeast; S, South; SE, Southeast). *Strain used for the virulence tests. [file Table1.DOCX]

| **Collection name** | **Isolation name** | **Location (Area)** | **Host** | **Sequevar** | **GenBank** |
| --- | --- | --- | --- | --- | --- |
| RUN2018 | YT13.130 | Benara mount (C) | *Capsicum frutescens* | 31 | MF359115 |
| RUN2165 | YT13.124 | Benara mount (C) | *Solanum melongena* | 31 | MF359158 |
| RUN2021 | YT13.126 | Benara mount (C) | *S. melongena* | 31 | MF359157 |
| RUN2157 | YT5.54 | Boudraguela (C) | *Solanum lycopersicum* | 31 | MF359161 |
| RUN2167 | YT5.56 | Boudraguela (C) | *S. lycopersicum* | 31 | MF359164 |
| RUN2162 | YT5.55 | Boudraguela (C) | *S. lycopersicum* | 31 | MF359159 |
| RUN2025 | YT5.58 | Boudraguela (C) | *S. lycopersicum* | 31 | MF359163 |
| RUN2152 | YT5.57 | Boudraguela (C) | *S. lycopersicum* | 31 | MF359162 |
| RUN2159 | YT5.59 | Boudraguela (C) | *S. lycopersicum* | 31 | MF359160 |
| RUN2170* | YT2.220 | Coconi (C) | *S. melongena* | 31 | MF359165 |
| RUN2172 | YT2.222 | Coconi (C) | *S. melongena* | 31 | MF359166 |
| RUN2137 | YT2.221 | Coconi (C) | *S. melongena* | 31 | MF359168 |
| RUN2032 | YT2.219 | Coconi (C) | *S. melongena* | 31 | MF359167 |
| RUN2140* | YT4.40 | Haboué (C) | *S. lycopersicum* | 15 | MF359102 |
| RUN2144 | YT4.42 | Haboué (C) | *S. lycopersicum* | 15 | MF359100 |
| RUN2054 | YT4.29 | Haboué (C) | *S. lycopersicum* | 31 | MF359193 |
| RUN2052 | YT4.30 | Haboué (C) | *S. lycopersicum* | 31 | MF359194 |
| RUN2083* | YT4.37 | Haboué (C) | *S. lycopersicum* | 18 | MF359114 |
| RUN2127* | YT4.38 | Haboué (C) | *S. lycopersicum* | 46 | MF359103 |
| RUN2143* | YT4.41 | Haboué (C) | *S. lycopersicum* | 15 | MF359095 |
| RUN2045 | YT4.33 | Haboué (C) | *S. lycopersicum* | 31 | MF359197 |
| RUN2051 | YT4.31 | Haboué (C) | *S. lycopersicum* | 31 | MF359195 |
| RUN2048 | YT4.32 | Haboué (C) | *S. lycopersicum* | 31 | MF359196 |
| RUN2024 | YT4.34 | Haboué (C) | *S. lycopersicum* | 18 | MF359108 |
| RUN2150* | YT4.36 | Haboué (C) | *S. lycopersicum* | 18 | MF359109 |
| RUN2146* | YT4.39 | Haboué (C) | *S. lycopersicum* | 46 | MF359104 |
| RUN2063 | YT4.43 | Haboué (C) | *S. lycopersicum* | 31 | MF359192 |
| RUN2068 | YT4.28 | Haboué (C) | *S. lycopersicum* | 31 | MF359190 |
| RUN2067 | YT4.35 | Haboué (C) | *S. lycopersicum* | 31 | MF359191 |
| RUN2029 | YT16.163 | Jitrolamhajou (C) | *Capsicum annuum* | 31 | MF359204 |
| RUN2027 | YT16.157 | Jitrolamhajou (C) | *S. lycopersicum* | 31 | MF359202 |
| RUN2030 | YT16.155 | Jitrolamhajou (C) | *S. melongena* | 31 | MF359154 |
| RUN2031 | YT16.153 | Jitrolamhajou (C) | *S. melongena* | 31 | MF359155 |
| RUN2033 | YT16.158 | Jitrolamhajou (C) | *S. melongena* | 31 | MF359156 |
| RUN2034 | YT16.160 | Jitrolamhajou (C) | *S. melongena* | 31 | MF359205 |
| RUN2035 | YT16.161 | Jitrolamhajou (C) | *S. melongena* | 31 | MF359206 |
| RUN2028 | YT16.154 | Jitrolamhajou (C) | *S. melongena* | 31 | MF359203 |
| RUN2041 | YT6.64 | Kagani (C) | *S. lycopersicum* | 31 | MF359211 |
| RUN2036 | YT6.67 | Kagani (C) | *S. lycopersicum* | 31 | MF359207 |
| RUN2037 | YT6.62 | Kagani (C) | *S. lycopersicum* | 31 | MF359208 |
| RUN2038 | YT6.61 | Kagani (C) | *S. lycopersicum* | 31 | MF359209 |
| RUN2039 | YT6.63 | Kagani (C) | *S. lycopersicum* | 31 | MF359210 |
| RUN2043 | YT3.26 | Kagani (C) | *S. lycopersicum* | 31 | MF359212 |
| RUN2132 | YT1.2 | Ongoujou (C) | *S. lycopersicum* | 31 | MF359140 |
| RUN2134 | YT1.10 | Ongoujou (C) | *S. lycopersicum* | 31 | MF359141 |
| RUN2135 | YT1.2bis | Ongoujou (C) | *S. lycopersicum* | 31 | MF359142 |
| RUN2136 | YT1.3 | Ongoujou (C) | *S. lycopersicum* | 31 | MF359143 |
| RUN2138 | YT1.11 | Ongoujou (C) | *S. lycopersicum* | 15 | MF359098 |
| RUN2158 | YT8.82 | Retenue collinaire (C) | *S. lycopersicum* | 31 | MF359150 |
| RUN2139 | YT8.81 | Retenue collinaire (C) | *S. lycopersicum* | 15 | MF359097 |
| RUN2156 | YT8.80 | Retenue collinaire (C) | *S. melongena* | 31 | MF359149 |
| RUN2141 | YT8.72 | Retenue collinaire (C) | *S. melongena* | 15 | MF359099 |
| RUN2142 | YT7.70 | Retenue collinaire (C) | *S. melongena* | 15 | MF359096 |
| RUN2151 | YT8.76 | Retenue collinaire (C) | *S. melongena* | 31 | MF359145 |
| RUN2153 | YT8.77 | Retenue collinaire (C) | *S. melongena* | 31 | MF359146 |
| RUN2154 | YT8.78 | Retenue collinaire (C) | *S. melongena* | 31 | MF359147 |
| RUN2155 | YT8.79 | Retenue collinaire (C) | *S. melongena* | 31 | MF359148 |
| RUN2145 | YT8.71 | Retenue collinaire (C) | *S. melongena* | 31 | MF359144 |
| RUN2147 | YT8.73 | Retenue collinaire (C) | *S. melongena* | 46 | MF359105 |
| RUN2148 | YT8.74 | Retenue collinaire (C) | *S. melongena* | 46 | MF359106 |
| RUN2149 | YT8.75 | Retenue collinaire (C) | *S. melongena* | 46 | MF359107 |
| RUN2166 | YT9.85 | Vahibé (C) | *S. lycopersicum* | 18 | MF359112 |
| RUN2169 | YT9.86 | Vahibé (C) | *S. lycopersicum* | 31 | MF359152 |
| RUN2171 | YT9.90 | Vahibé (C) | *S. lycopersicum* | 31 | MF359153 |
| RUN2163 | YT9.88 | Vahibé (C) | *S. lycopersicum* | 18 | MF359111 |
| RUN2164 | YT9.87 | Vahibé (C) | *S. lycopersicum* | 18 | MF359113 |
|  |  |  |  |  |  |
|  |  |  |  |  |  |
|  |  |  |  |  |  |
| **Collection name** | **Isolation name** | **Location (Area)** | **Host** | **Sequevar** | **Genbank** |
| RUN2100 | YT18.176 | Dzoumognyé (N) | *C. frutescens* | 31 | MF359181 |
| RUN2095 | YT17.170 | Dzoumognyé (N) | *S. lycopersicum* | 31 | MF359183 |
| RUN2098 | YT18.172 | Dzoumognyé (N) | *S. melongena* | 31 | MF359182 |
| RUN2042 | YT18.171 | Dzoumognyé (N) | *S. melongena* | 31 | MF359186 |
| RUN2073 | YT18.174 | Dzoumognyé (N) | *S. melongena* | 31 | MF359188 |
| RUN2071 | YT18.175 | Dzoumognyé (N) | *S. melongena* | 31 | MF359189 |
| RUN2091 | YT17.166 | Dzoumognyé (N) | *S. melongena* | 31 | MF359184 |
| RUN2085 | YT17.168 | Dzoumognyé (N) | *S. melongena* | 31 | MF359185 |
| RUN2075 | YT18.173 | Dzoumognyé (N) | *S. melongena* | 31 | MF359187 |
| RUN2113 | YT21.193 | Miangani (N) | *S. lycopersicum* | 31 | MF359130 |
| RUN2109 | YT21.190 | Miangani (N) | *S. lycopersicum* | 31 | MF359126 |
| RUN2110 | YT20.189 | Miangani (N) | *S. lycopersicum* | 31 | MF359127 |
| RUN2111 | YT21.191 | Miangani (N) | *S. lycopersicum* | 31 | MF359128 |
| RUN2112 | YT21.192 | Miangani (N) | *S. lycopersicum* | 31 | MF359129 |
| RUN2097 | YT21.195 | Miangani (N) | *S. lycopersicum* | 31 | MF359125 |
| RUN2115 | YT19.184 | Mitséni (N) | *C. annuum* | 31 | MF359132 |
| RUN2122 | YT19.180 | Mitséni (N) | *S. melongena* | 31 | MF359135 |
| RUN2119 | YT19.179 | Mitséni (N) | *S. melongena* | 31 | MF359134 |
| RUN2123 | YT19.182 | Mitséni (N) | *S. melongena* | 31 | MF359136 |
| RUN2124 | YT19.183 | Mitséni (N) | *S. melongena* | 31 | MF359137 |
| RUN2114 | YT19.181 | Mitséni (N) | *S. melongena* | 31 | MF359131 |
| RUN2117 | YT19.185 | Mitséni (N) | *S. melongena* | 31 | MF359133 |
| RUN2079 | YT22.199 | Kaweni (NE) | *S. lycopersicum* | 31 | MF359233 |
| RUN2070 | YT22.196 | Kaweni (NE) | *S. lycopersicum* | 31 | MF359231 |
| RUN2072 | YT22.198 | Kaweni (NE) | *S. lycopersicum* | 31 | MF359232 |
| RUN2084 | YT22.201 | Kaweni (NE) | *S. lycopersicum* | 31 | MF359234 |
| RUN2066 | YT22.197 | Kaweni (NE) | *S. lycopersicum* | 31 | MF359229 |
| RUN2086 | YT22.202 | Kaweni (NE) | *S. melongena* | 31 | MF359116 |
| RUN2087 | YT22.205 | Kaweni (NE) | *S. melongena* | 31 | MF359117 |
| RUN2064 | YT23.206 | Kaweni (NE) | *S. melongena* | 31 | MF359227 |
| RUN2065 | YT22.203 | Kaweni (NE) | *S. melongena* | 31 | MF359228 |
| RUN2069 | YT23.207 | Kaweni (NE) | *S. melongena* | 31 | MF359230 |
| RUN2094 | YT24.215 | Kaweni (NE) | *S. lycopersicum* | 31 | MF359123 |
| RUN2089 | YT24.212 | Kaweni (NE) | *S. melongena* | 31 | MF359119 |
| RUN2090 | YT24.209 | Kaweni (NE) | *S. melongena* | 31 | MF359120 |
| RUN2092 | YT24.210 | Kaweni (NE) | *S. melongena* | 31 | MF359121 |
| RUN2093 | YT24.214 | Kaweni (NE) | *S. melongena* | 31 | MF359122 |
| RUN2096 | YT24.217 | Kaweni (NE) | *Solanum nigrum* | 31 | MF359124 |
| RUN2088 | YT24.216 | Kaweni (NE) | *S. nigrum* | 31 | MF359118 |
| RUN2053 | YT12.112 | Kani Keli (S) | *S. lycopersicum* | 31 | MF359218 |
| RUN2055 | YT12.113 | Kani Keli (S) | *S. lycopersicum* | 31 | MF359219 |
| RUN2056 | YT12.114 | Kani Keli (S) | *S. lycopersicum* | 31 | MF359220 |
| RUN2057 | YT12.115 | Kani Keli (S) | *S. lycopersicum* | 31 | MF359221 |
| RUN2058 | YT12.117 | Kani Keli (S) | *S. lycopersicum* | 31 | MF359222 |
| RUN2059 | YT12.118 | Kani Keli (S) | *S. lycopersicum* | 31 | MF359223 |
| RUN2060 | YT12.119 | Kani Keli (S) | *S. lycopersicum* | 31 | MF359224 |
| RUN2061 | YT12.121 | Kani Keli (S) | *S. lycopersicum* | 31 | MF359225 |
| RUN2062 | YT12.122 | Kani Keli (S) | *S. lycopersicum* | 31 | MF359226 |
| RUN2044 | YT12.120 | Kani Keli (S) | *S. lycopersicum* | 31 | MF359213 |
| RUN2046 | YT12.116 | Kani Keli (S) | *S. lycopersicum* | 31 | MF359214 |
| RUN2047 | YT12.110 | Kani Keli (S) | *S. lycopersicum* | 31 | MF359215 |
| RUN2049 | YT12.109 | Kani Keli (S) | *S. lycopersicum* | 31 | MF359216 |
| RUN2050 | YT12.111 | Kani Keli (S) | *S. lycopersicum* | 31 | MF359217 |
| RUN2116 | YT15.144 | Dembeni (SE) | *C. annuum* | 31 | MF359176 |
| RUN2108* | YT15.145 | Dembeni (SE) | *C. annuum* | 31 | MF359177 |
| RUN2131 | YT15.149 | Dembeni (SE) | *C. annuum* | 31 | MF359170 |
| RUN2107 | YT15.148 | Dembeni (SE) | *C. annuum* | 31 | MF359178 |
| RUN2103 | YT15.150 | Dembeni (SE) | *C. annuum* | 31 | MF359179 |
| RUN2121 | YT15.141 | Dembeni (SE) | *S. lycopersicum* | 31 | MF359173 |
| RUN2129 | YT15.139 | Dembeni (SE) | *S. lycopersicum* | 31 | MF359171 |
| RUN2118 | YT15.143 | Dembeni (SE) | *S. lycopersicum* | 31 | MF359175 |
| RUN2040 | YT15.140 | Dembeni (SE) | *S. lycopersicum* | 31 | MF359172 |
| RUN2120 | YT15.142 | Dembeni (SE) | *S. lycopersicum* | 31 | MF359174 |
| RUN2133 | YT15.151 | Dembeni (SE) | *S. nigrum* | 31 | MF359169 |
| RUN2102 | YT15.152 | Dembeni (SE) | *S. nigrum* | 31 | MF359180 |
| RUN2026 | YT10.93 | Ironi bé (SE) | *S. lycopersicum* | 31 | MF359201 |
| RUN2019 | YT10.94 | Ironi bé (SE) | *S. lycopersicum* | 31 | MF359198 |
| RUN2020 | YT10.92 | Ironi bé (SE) | *S. lycopersicum* | 31 | MF359199 |
| RUN2022 | YT10.91 | Ironi bé (SE) | *S. lycopersicum* | 31 | MF359200 |
| RUN2126 | YT11.107 | M'romouhou (SE) | *C. frutescens* | 31 | MF359139 |
| RUN2125 | YT11.98 | M'romouhou (SE) | *S. lycopersicum* | 31 | MF359138 |
| RUN2130 | YT11.102 | M'romouhou (SE) | *S. melongena* | 15 | MF359101 |
| RUN2160 | YT14.138 | Tsararano (SE) | *S. lycopersicum* | 31 | MF359151 |
| RUN2161 | YT14.133 | Tsararano (SE) | *S. lycopersicum* | 18 | MF359110 |

^a^ Mayotte location and area of isolated strains (C = Center, N = North, NE = North-Est, S = South, SE = South-Est).

*****Strains used for the pathogenicity test.
