# Supplementary material for: Sequevar Diversity and Virulence of Ralstonia solanacearum Phylotype I on Mayotte Island (Indian Ocean)
Source: Front Plant Sci. 2018 Jan 5;8:2209. doi: 10.3389/fpls.2017.02209 (PMC5760537; doi:10.3389/fpls.2017.02209)
Supplement: Table S2 — RSSC reference strains used in this study to structure the phylogenetic tree. [file Table2.DOCX]

| **Strain** | **Alternative name,**  **GenBank** | **Host** | **Origin** | **Phylotype - Sequevar** | |  |
| --- | --- | --- | --- | --- | --- | --- |
| A3909 | UW628, EF371812 | *Heliconia rostrata* | Usa | IIA-6 | |  |
| CFBP2957 | MT5, AF295265 | *Solanum lycopersicum* | Martinique | IIA-36 |  | |
| CFBP2958 | GT4, AF295266 | *Solanum lycopersicum* | Guadeloupe | IIA-39 |  | |
| CFBP2972 | MPT1, AF295264 | *Solanum tuberosum* | Martinique | IIA-35 |  | |
| CIP301 | R311, GU295003 | *Solanum tuberosum* | Peru | IIA-35 |  | |
| ICMP7963 | UW134, AF295263 | *Solanum tuberosum* | Kenya | IIA-7 |  | |
| CFBP2047 | K60, AF295262 | *Solanum lycopersicum* | Usa | IIA-7 |  | |
| UW469 | CIP239, DQ657612 | *Solanum tuberosum* | Brazil | IIA-40 |  | |
| CFBP7032 | CMR39, EF439726 | *Solanum lycopersicum* | Cameroon | IIA-41 |  | |
| CFBP7054 | CMR121, EF439725 | *Solanum lycopersicum* | Cameroon | IIA-52 |  | |
| CFBP6779 | 02-174, EF371872 | *Canna indica* | Martinique | IIA-38 |  | |
| IBSBF1900 | RUN301, EF371839 | *Musa* sp. | Brazil | IIA-24 |  | |
| T1-UY | RUN448, GU295049 | *Solanum lycopersicum* | Uruguay | IIA-50 |  | |
| UW181 | CFBP6438, GU295053 | *Banana plantain* | Venezuela | IIA-6 |  | |
| RF27 | RUN543, EU726804 | *Solanum lycopersicum* | Trinidad | IIA-35 |  | |
| GMI8044 | BA7, GU295013 | *Banana* | Grenada | IIA-6 |  | |
| CFBP6783 | ANT75, EF371817 | *Heliconia caribea* | Martinique | IIB-4NPB |  | |
| IPO1609 | UW627, EF371814 | *Solanum tuberosum* | Netherlands | IIB-1 |  | |
| CIP10 | UW477, AF295260 | *Solanum tuberosum* | Peru | IIB-25 |  | |
| CIP117 | NCPPB2088, JF702301 | *Solanum tuberosum* | Nigeria | IIB-1 |  | |
| MOLK2 | R633, EF371841 | *Musa sp.* | Philippines | IIB-3 |  | |
| NCPPB3987 | R590, AF295261 | *Solanum tuberosum* | Brazil | IIB-28 |  | |
| UW70 | S210, DQ011550 | *Banana plantain* | Colombia | IIB-4 |  | |
| JT516 | RUN160, AF295258 | *Solanum tuberosum* | Reunion | IIB-1 |  | |
| CIP231 | EF647734 | *Solanum tuberosum* | Chile | IIB-2 |  | |
| CIP418 | Moh6, GU295005 | *Arachis hypogaea* | Indonesia | IIB-3 |  | |
| CFBP7029 | CMR34, EF439750 | *Solanum lycopersicum* | Cameroon | IIB-1 |  | |
| CFBP7014 | GMI8291, AF371831 | *Anthurium andreanum* | Trinidad | IIB-51 |  | |
| IBSBF1712 | RUN299, EF371833 | *Pelargonium asperum* | Brazil | IIB-27 |  | |
| CIP240 | JT662, EF647739 | *Solanum tuberosum* | Brazil | IIB-26 |  | |
| UW163 | CFBP1419, GU295052 | *Banana plantain* | Peru | IIB-4 |  | |
| CFBP3059 | JS904, AF295270 | *Solanum melongena* | Burkina Faso | III-23 |  | |
| J25 | RUN56, AF295279 | *Solanum tuberosum* | Kenya | III-20 |  | |
| JT525 | UW647, AF295272 | *Pelargonium asperum* | Reunion | III-19 |  | |
| NCPPB0332 | JS949, AF295276 | *Solanum tuberosum* | Zimbabwe | III-22 |  | |
| NCPPB0342 | CFBP6430, JF702305 | *Nicotinia tabacum* | Zimbabwe | III-20 |  | |
| CMR15 | CFBP6941, JF702319 | *Solanum lycopersicum* | Cameroon | III-29 |  | |
| CFBP6942 | CMR32, EF439749 | *Solanum scabrum* | Cameroon | III-29 |  | |
| CFBP7038 | CMR66, EF439729 | *Solanum scabrum* | Cameroon | III-49 |  | |
| DGBBC1138 | RUN362, GU295009 | *Solanum tuberosum* | Guinea | III-43 |  | |
| DGBBC1227 | RUN364, GU295011 | *Solanum tuberosum* | Guinea | III-42 |  | |
| DGBBC1125 | RUN369, GU295008 | *Solanum tuberosum* | Guinea | III-43 |  | |
| NCPPB1018 | JS950, AF295271 | *Solanum tuberosum* | Angola | III-21 |  | |
| ACH732 | CIP357, GQ907150 | *Solanum lycopersicum* | Australia | IV-11 |  | |
| R230 | UW648, JF702303 | *Musa* sp. | Indonesia | IV-10 |  | |
| JT663 | R008, JF702304 | *Syzygium aromaticum* | Indonesia | IV-9A |  | |
| MAAF301558 | JS394, DQ011558 | *Solanum tuberosum* | Japan | IV-8 |  | |
| PSI07 | CFBP7288, EF371804 | *Solanum lycopersicum* | Indonesia | IV-10 |  | |
| R24 | UQRS466, JF702321 | *Syzygium aromaticum* | Indonesia | IV -9 |  | |
