# Supplementary material for: Sequevar Diversity and Virulence of Ralstonia solanacearum Phylotype I on Mayotte Island (Indian Ocean)
Source: Front Plant Sci. 2018 Jan 5;8:2209. doi: 10.3389/fpls.2017.02209 (PMC5760537; doi:10.3389/fpls.2017.02209)
Supplement: Table S3 — Tomato accessions used for the virulence tests. [file Table3.DOCX]

| Code | Accession | Species | Seed sources | Assays | |
| --- | --- | --- | --- | --- | --- |
|  |  |  |  | Field | Controlled |
| T1  T2  T3  T4  T5  T6  T7  T8  T9  T10  P  C | CRA66  Okitsu Sozai  NC 72 TR 4-4  IRAT L3  Hawaii 7996  TML46  CLN1463  R3034  L285  L390  Platinium F1  Cobra F1 | *Solanum lycopersicum* var.*cerasiforme*  *S. lycopersicum*  *S.lycopersicum*  *S. lycopersicum*  *S. lycopersicum*  *S. lycopersicum*  *S. lycopersicum*  *S. lycopersicum*  *Solanum lycopersicum* var.*cerasiforme*  *S. lycopersicum* var.*cerasiforme*  *S. lycopersicum*  *S. lycopersicum* | INRA  INRA  INRA  INRA  INRA  AVRDC  AVRDC  AVRDC  AVRDC  AVRDC  East West Seeds  Technisem | X  X  X  X  X  X  X  X  X  X | X  X  X  X  X  X  X  X  X |
