# Supplementary material for: Sequevar Diversity and Virulence of Ralstonia solanacearum Phylotype I on Mayotte Island (Indian Ocean)
Source: Front Plant Sci. 2018 Jan 5;8:2209. doi: 10.3389/fpls.2017.02209 (PMC5760537; doi:10.3389/fpls.2017.02209)
Supplement: Table S4 — Sequevar distributions of the Ralstonia solanacearum phylotype I strains within the five vegetable production areas of Mayotte. [file Table4.DOCX]

| Area | No. of strains  Phylotype-sequevar of *R. pseudosolanacearum* | | | | Total no. of strains (%) |
| --- | --- | --- | --- | --- | --- |
|  | I- 31 | I-18 | I-46 | I-15 |  |
| North | 22 (100.0%) | - | - | - | 22 (15.7%) |
| North-East | 17 (100.0%) | - | - | - | 17 (12.1%) |
| Center | 48 (72.7%) | 6 (9.1%) | 5 (7.6%) | 7 (10.6%) | 66 (47.1%) |
| South | 14 (100.0%) | - | - | - | 14 (10.0%) |
| South-East | 19 (90.5%) | 1 (4.8%) | - | 1 (4.8%) | 21 (15.0%) |
| Total no. of strains (%) | 120 (85.7%) | 7 (5.0%) | 5 (3.6%) | 8 (5.7%) | 140 (100.0%) |
