# Supplementary material for: Sequevar Diversity and Virulence of Ralstonia solanacearum Phylotype I on Mayotte Island (Indian Ocean)
Source: Front Plant Sci. 2018 Jan 5;8:2209. doi: 10.3389/fpls.2017.02209 (PMC5760537; doi:10.3389/fpls.2017.02209)
Supplement: Table S5 — Sequevar distributions of the Ralstonia solanacearum phylotype I strains within the different host species sampled in Mayotte. [file Table5.DOCX]

| Host species | No. of strains  Phylotype-sequevar of *R. pseudosolanacearum* | | | | Total |
| --- | --- | --- | --- | --- | --- |
|  | I- 31 | I-18 | I-46 | I-15 |  |
| Hot pepper | 2 (66.7%) | 1 (33.3%) | 0 (0.0%) | 0 (0.0%) | 3 (2.1%) |
| Sweet pepper | 5 (71.4%) | 0 (0.0%) | 1 (14.3%) | 1 (14.3%) | 7 (5.0%) |
| Tomato | 73 (91.3%) | 3 (3.8%) | 4 (5.0%) | 0 (0.0%) | 80 (57.1%) |
| Eggplant | 37 (80.4%) | 2 (4.3%) | 0 (0.0%) | 7 (15.2%) | 46 (32.9%) |
| Blacknightshade | 3 (75.0%) | 1 (25.0%) | 0 (0.0%) | 0 (0.0%) | 4 (2.9%) |
| Total | 120 (85.7%) | 7 (5.0%) | 5 (3.6%) | 8 (5.7%) | 140 (100.0%) |
