# Supplementary material for: Sequevar Diversity and Virulence of Ralstonia solanacearum Phylotype I on Mayotte Island (Indian Ocean)
Source: Front Plant Sci. 2018 Jan 5;8:2209. doi: 10.3389/fpls.2017.02209 (PMC5760537; doi:10.3389/fpls.2017.02209)
Supplement: Table S6 — Comparison table of the mean wilted rate of several tomato lines from the Core collection measured under field and controlled conditions against Ralstonia solanacearum phylotype I-31 strains from Mayotte. aTomato accessions from the tomato Core-Tep. bField experiment was assessed on the tomato Core-tep in Mayotte in 2011 and screened against strains characterized as R. pseudosolanacearum phylotype I sequevar 31. Notably, the RUN2108 (Solanum lycopersicum), RUN2116 (Capsicum annuum), and RUN2121 (Solanum nigrum) strains were collected in the same tunnel in 2012. cScreening results obtained for strain RUN2108 (I-31), which was collected in the same tunnel in 2012 against several tomato Core-Tep lines under controlled conditions in La Reunion in 2013. [file Table6.DOCX]

| *R. solanacearum* strains from Mayotte (phylotype-sequevar) | | |
| --- | --- | --- |
| Lines^a^ | Tested under field conditions^b^ | Tested under controlled conditions^c^ |
|  | W | W |
| T1 | 80.0 | - |
| T2 | 78.1 | - |
| T3 | 72.5 | - |
| T4 | 63.3 | 56.0 |
| T5 | 9.2 | 10.0 |
| T6 | 2.5 | - |
| T7 | 15 | 16.7 |
| T8 | 2.5 | 3.3 |
| T9 | 32.5 | 40.0 |
| T10 | 100 | 100.0 |
